# Agenesis of the dorsal pancreas with chronic suppurative pancreatitis: Case report and literature review

**DOI:** 10.1097/MD.0000000000028137

**Published:** 2021-12-10

**Authors:** Lei-Zhou Xia, Xue-Feng Bu, Peng-Cheng Jiang, Feng Yu, Yong-Jun Zhang, Na-Na Meng

**Affiliations:** a Department of General Surgery, Affiliated People's Hospital, Jiangsu University, Zhenjiang, Jiangsu Province, China; b Department of Ophthalmology, Affiliated People's Hospital, Jiangsu University, Zhenjiang, Jiangsu Province, China; c Department of Ophthalmology, Zhenjiang Kangfu Eye Hospital, Zhenjiang, Jiangsu Province, China.

**Keywords:** agenesis of the dorsal pancreas, case report, diabetes mellitus, pancreatic anomaly, pancreatitis

## Abstract

**Rationale:**

Agenesis of the dorsal pancreas (ADP) is a rare congenital anomaly of the pancreas. ADP is associated with some other medical problems such as diabetes mellitus, abdominal pain/bloating, pancreatitis, pancreatic neuroendocrine tumor and so on. In this study, we present a case of ADP with chronic suppurative pancreatitis, summarize the clinical characteristics of the reported cases in China and review the correlative literature.

**Patient concerns:**

A 51-year-old Chinese man, with a history of impaired fasting glucose, presented with jaundice, pruritus and dark urine. Laboratory analysis showed abnormal liver function and elevated carbohydrate antigen 19-9.

**Diagnoses:**

Contrast-enhanced computed tomography demonstrated a mass located at the head of pancreas and complete absence of the body and tail of pancreas. Endoscopic retrograde cholangiopancreatography demonstrated an eccentric malignant stricture about 1.6cm of distal common bile duct.

**Interventions:**

The patient underwent pancreaticoduodenectomy because of the suspicion of pancreatic tumor. The postoperative pathological result was chronic suppurative pancreatitis, with moderate hyperplasia in focal ductal epithelium.

**Outcomes:**

A long-term follow-up shows that the patient is asymptomatic with well-controlled diabetes mellitus and pancreatic exocrine insufficiency.

**Lessons:**

ADP is a quite rare congenital malformation of the pancreas with poorly-understood pathogenesis. The diagnosis of ADP depends on the imaging examination. The therapeutic strategy varies from person to person due to the different accompanying conditions.

## 1 Introduction

The pancreas develops from dorsal and ventral pancreatic buds arising from the caudal region of the embryonic foregut. The dorsal pancreatic bud contributes to the upper part of the head, body and tail of the pancreas, and the ventral pancreatic bud eventually develops into the uncinate process and inferior part of the head of the pancreas. Agenesis of the dorsal pancreas (ADP), also known as congenital short pancreas, is a rare congenital abnormality resulted from the embryological failure of the dorsal pancreatic bud to form the body and tail of the pancreas.^[1,2]^ Up to now, around 100 cases of ADP have been reported worldwide since 191,^[3,4]^ whereas <20 cases have been reported in China as we know so far.^[5–19]^ Here we reported a a case of ADP with chronic suppurative pancreatitis and evaluated the clinical features of patients with ADP in China.

## 2 Case presentation

A 51-year-old man was admitted to the hospital with a 10-day history of jaundice, pruritus and dark urine. There were no other complaints. The patient underwent cholecystectomy for cholecystolithiasis in another medical unit nine months ago. He complained of impaired fasting glucose for several months yet did not confirm the diagnosis of diabetes mellitus (DM). He had no history of other diseases, and the family history was unremarkable. On admission, the abdomen was soft, not distended, and the patient presented no painful abdomen on palpation, no abdominal guarding and no palpable masses. Physical examination was otherwise within normal limits.

**Figure 1:**
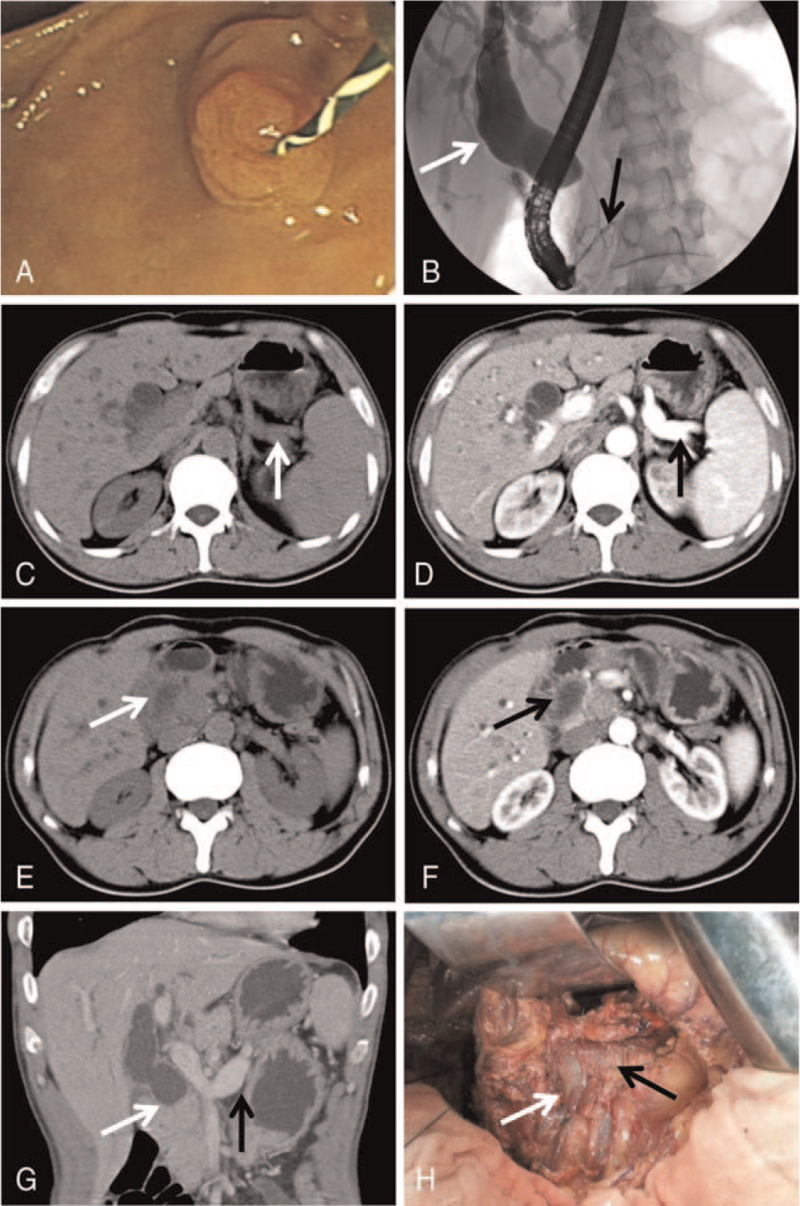
Endoscopic retrograde cholangiopancreatography (ERCP) images, abdominal CT imaging and intraoperative image of the reported ADP case. (A) Major duodenal papilla by duodenoscopy. (B) ERCP showing dilated common bile duct and the location of the bile duct obstruction (white arrow), and the pancreatic duct located at the head of the pancreas (black arrow). Non-contrast CT (C) and contrast-enhanced CT (D) showed splenic vessels (C, white arrow; D, black arrow) and complete absence of the body and tail of pancreas. Non-contrast CT (E) and contrast-enhanced CT (F) showed a mass located at pancreas head with dilation of common bile duct (E, white arrow; F black arrow). Coronal CT scanning (G) showed the pancreas head and dilated common bile duct (white arrow), and the splenic vein with absence of the body and tail of pancreas (black arrow). Intraoperative image (H) showed superior mesenteric vein (white arrow), and the splenic vein with absence of the body and tail of pancreas (black arrow).

**Figure 2:**
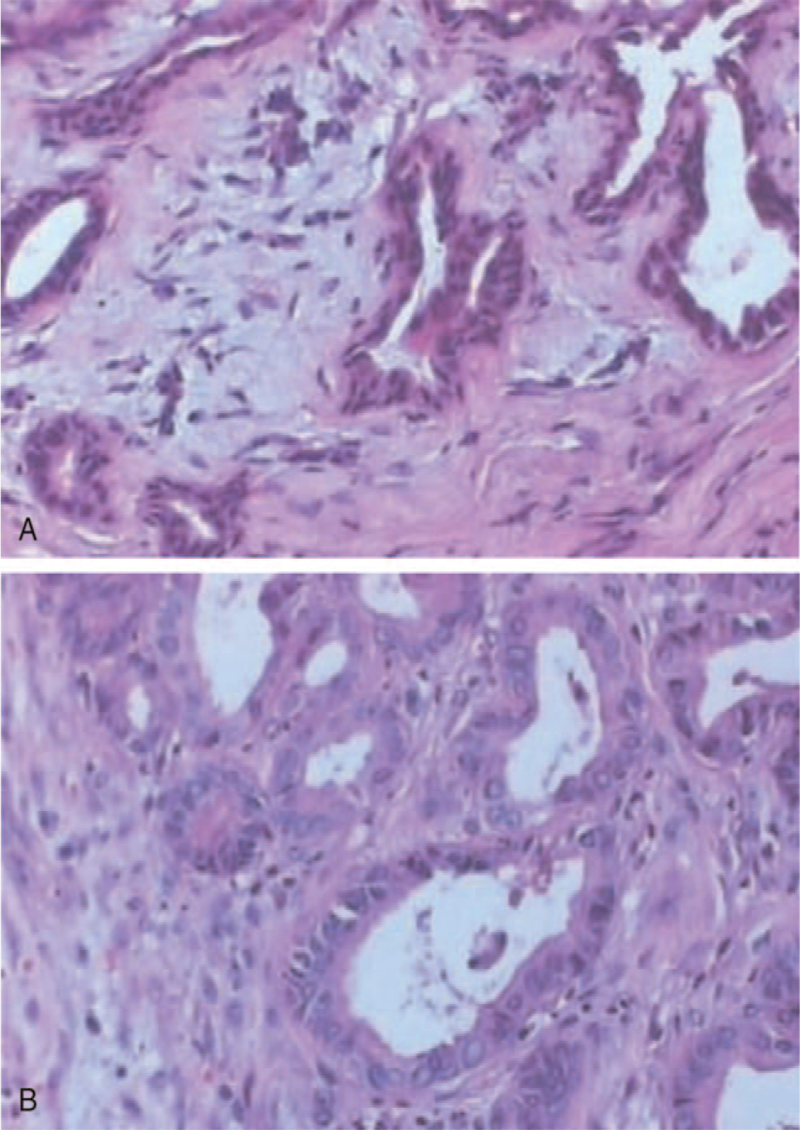
Representative HE staining of pancreatic tissue images from the reported case of ADP (×20 objective).

Laboratory assessment revealed: leukocytes 6.0 × 10^9^ cell/L (reference values from 4.0∼10.0 × 10^9^ cells/L), total bilirubin 139.5 μmol/L (2.1∼17.3), direct bilirubin 55.1 μmol/L (0∼5.8), alanine aminotransferase 1822 U/L (0∼40), alkaline phosphatase 442 U/L (40∼135), γ-glutamyl transpeptidase 378.1 U/L (5∼40), and both serum amylase and lipase were within the normal range, carbohydrate antigen 19–9 103.1 U/mL (0∼30.0), carbohydrate antigen 24-2 143 U/mL (0∼25.0), carbohydrate antigen 50 160 U/mL (0∼20.0). An abdominal computed tomography (CT) scan showed a mass located at the pancreas head with the dilation of intrahepatic and extrahepatic bile duct, and complete absence of the body and tail of pancreas (Fig. 1C–G). Major duodenal papilla was normal by electronic duodenoscopy (Fig. 1A). Endoscopic retrograde cholangiopancreatography (ERCP) demonstrated an eccentric malignant stricture about 1.6 cm of distal common bile duct. Figure 1B showed remarkably dilated common bile duct, intrahepatic bile duct and the location of the bile duct obstruction as well. Moreover, only the pancreatic duct with normal diameter located at the head of the pancreas was demonstrated. Combined with the analysis of the imaging examination results, abnormal liver function and elevated tumor marker levels, carcinoma of head of pancreas suspected. With thorough preoperative discussion and communication with the patient's family, the patient underwent a laparotomy operation, during which a mass located at the pancreas head, the absence of the body and tail of pancreas was confirmed (Fig. 1F). Intraoperative frozen section examination was conducted twice. The first result showed no obvious atypia in the inspected pancreas tissue, yet the subsequent result was chronic suppurative pancreatitis, with moderate hyperplasia in focal ductal epithelium. The surgery was continued after adequate communication with the patient's family. The gallbladder, common bile duct, duodenum, and the pancreas head were firstly resected as the routine pylorus-preserving Whipple procedure. Then choledojejunostomy and gastrojejunostomy were performed routinely. In the present case, this procedure was almost equivalent to total pancreatectomy. Thus, there was no pancreaticojejunostomy or the other pancreatic drainage routes. The postoperative pathological result was chronic suppurative pancreatitis, with moderate hyperplasia in focal ductal epithelium as well. Figure 2 shows the representative hematoxylin-eosin staining of pancreatic tissue images. Generally, the blood glucose of the patient was under control during the perioperative period. Fasting blood glucose values (venous blood) after operation were shown as follows: 1st day 25.54 mmol/L, 3rd day 9.73 mmol/L, 5th day 8.96 mmol/L, and 14th day 8.94 mmol/L. Moreover, the patient's postoperative glucose values of peripheral blood were monitored every 4 hours, fluctuating from approximately 13.8 to 24.8 mmol/L during the first 24 hours after the operation and 4.5 to 17.8 mmol/L during the 2nd day to the 14th day after the surgery. Parenteral nutrition was performed for 10 days after the operation with the ratio of neutral insulin (U) to glucose (g) 1:4. Meanwhile, neutral insulin pump including 50 mL normal saline and 50 U neutral insulin was applied continuously to control the perioperative blood glucose level. The pump speed was adjusted according to the glucose value of peripheral blood. With appropriate postoperative treatment, the patient was asymptomatic and discharged two weeks after the operation. As a whole, the therapeutic strategy for this patient was aggressive, yet appropriate to some extent, considering the final diagnosis (chronic suppurative pancreatitis, with moderate hyperplasia in focal ductal epithelium), which was the precancerous lesion of the pancreas and could develop into pancreatic cancer with high probability. A long-term follow-up with 7 years was performed. Now the patient is asymptomatic with well-controlled DM by subcutaneous injection of insulin aspart 16 IU before breakfast and 10 IU before dinner and takes 0.6 g pancreatin enteric-coated capsules before each meal as the pancreatic exocrine function supplement. Unfortunately, the periodic reexamination of imaging was not carried out.

## 3 Discussion

The embryological development of the pancreas is a complex process, during which the dorsal pancreatic bud forms the body and the tail of the pancreas, and gives rise to the accessory pancreatic duct, which is also called duct of Santorini.^[2,20]^ Any failure in the development of the dorsal bud therefore leads to an absence of a functional pancreatic body, tail, and duct of Santorini. This anomaly could be partial or complete. In partial ADP, the minor papilla, duct of Santorini, or the pancreatic body are present. In complete ADP, the neck, the body, and the tail of the pancreas, duct of Santorini, and minor papilla are all absent.^[21]^

The exact genetic pathogenesis of ADP is still poorly understood. *Hepatocyte Nuclear Factor 1-Beta* and *GATA Binding Protein 6* genes were proven to be correlated with the embryogenic development of the pancreas.^[22,23]^ Besides, experiments in mice showed that *homeobox protein HB9* gene and *retinaldehyde dehydrogenase 2* gene mutation or deficiency resulted in ADP.^[24,25]^ Consistent results, however, were not observed in humans.

According to the study of Schnedl et al, only 53 cases of ADP have been reported from 1911 to 2008.^[3]^ Most ADP patients were asymptomatic, yet commonly associated with DM and other additional medical problems such as pancreatitis, abdominal pain, polysplenia syndrome, and so on. A recent study further summarized the demographics of 53 cases of ADP in the published studies of the Medline and ISI Web of Science Databases from 2008 to 2015.^[4]^ Except for the common associated diseases like DM and pancreatitis, it described 9 cases of pancreatic adenocarcinoma, who underwent total pancreatectomy.

Due to the language reasons, the aforementioned systematic reviews did not include all the ADP cases in China. In the present study, we performed a literature search using the terms “agenesis of the dorsal pancreas,” “dorsal pancreatic agenesis,” “congenital short pancreas” from the Medline and Chinese databases-CNKI, Wanfang, Baidu Scholar. Overall, 15 articles and 18 cases of ADP (including the present case) were included in this literature review. The clinical features of 17 cases reported in Chinese region were summarized in Table 1.^[5–19]^ These cases aged from 15 to 67 and comprised 5 males and 13 females, among whom one case was confirmed the diagnosis by autopsy.^[6]^ The first case of ADP was also incidentally found by autopsy in 1911.^[26]^ In Chinese cases, most cases (11/18) had DM or impaired glucose tolerance, which was similar to the previous reviews.^[3,4]^ Most of the islet cells locate at the body and tail of the pancreas, thus ADP contributes to the development of DM.^[27]^ Abdominal pain is another common presenting symptom with this anomaly, yet it may be resulted from other diseases like pancreatitis or pancreatic tumor in some patients with ADP. In our review, abdominal pain was detected in 7 of 18 cases of ADP. Of note, gastrointestinal malrotation was described in 3 of 18 Chinese ADP cases. It is regarded that the dorsal and ventral pancreatic buds fuse during rotation of the gut tube at the seventh week of gestation,^[3,4]^ which may cause the linkage between the pancreatic anomaly and gastrointestinal malrotation. The pathological result showed that our ADP case was associated with chronic suppurative pancreatitis, with moderate hyperplasia in focal ductal epithelium of the pancreas, which was actually considered to be the precancerous lesion of the pancreas. Previous studies have confirmed that ADP patients may combine with chronic pancreatitis and periampullary tumors including pancreatic adenocarcinoma, neuroendocrine tumor, solid pseudopapillary tumor of the pancreas, and cholangiocarcinoma.^[18,28–31]^ Cholangiocarcinoma and solid pseudopapillary tumor of the pancreas were described to be combined with Chinese ADP patients in our review as well.^[8,10]^

**Table 1:** Characteristics of reported cases of dorsal pancreas agenesis in China.

Case	Author/year (reference)	Age, y/sex	Symptoms	Family history	Diagnostic method	Type	Associated diseases
1	Wang et al, 1990^[5]^	54/M	Abdominal pain	NA	Ultrasound, ERCP, CT, Surgery	Complete	DM, Dilated biliary trees
2	Lao and Mo, 2002^[6]^	15/F	NA	NA	Autopsy	Complete	NA
3	Ma and Li, 2003^[7]^	35/F	Epigastric pain, fever, jaundice	NA	Ultrasound, CT	Complete	Cholelithiasis
4	Yi et al, 2003^[8]^	58/F	Jaundice, low fever	NA	ERCP, MR, surgery	Complete	D, Intestinal malrotation, Distal cholangiocarcinoma
5	Du et al, 2007^[9]^	43/F	Epigastric pain, vomit, diarrhea, weight loss	Unremarkable	CT, MR	Complete	DM
6	Wei and Sun, 2009^[10]^	23/F	Unremarkable	NA	CT, Surgery	Partial	Solid-pseudopapillary tumor of the pancreas
7	Lin and Chen, 2013^[11]^	35/M	Unremarkable	Unremarkable	CT	Partial	DM
8	Zhou et al, 2014^[12]^	56/M	Unremarkable	NA	CT	Complete	Unremarkable
9	Li et al, 2015^[13]^	23/F	Unconsciousness	Unremarkable	CT	Complete	DM, Metabolic acidosis
10	Wu, 2015^[14]^	32/F	Unremarkable	NA	CT	Complete	Polysplenia, Gastric malrotation
11		52/F	Abdominal pain	NA	CT	Partial	Acute pancreatitis, Polysplenia
12	Zheng and He, 2015^[15]^	51/F	Unremarkable	NA	CT	Complete	Gastric volvulus, Intestinal malrotation
13	Liang et al, 2018^[16]^	23/F	Hyperglycemia	Unremarkable	MR	Complete	DM
14	Yang et al, 2019^[17]^	30/M	Epigastric pain	Unremarkable	CT, MR	Complete	Diabetic ketoacidosis
15	Mei et al, 2020^[18]^	65/F	Abdominal pain, nausea, bloating, acid regurgitation	Unremarkable	CT	Complete	DM
16		61/F	Upper right abdominal cramping, vomit, fever	Unremarkable	CT	Complete	DM, Gallbladder stone, Intermittent bloating after meals
17	Zhong et al, 2020^[19]^	67/F	Lower back pain	NA	CT, MR, endoscopic ultrasound	Complete	Impaired glucose tolerance
18	Present case	51/M	Jaundice, pruritus, dark urine	NA	ERCP, CT, surgery	Complete	DM, Chronic suppurative pancreatitis, Moderate hyperplasia in focal ductal epithelium of the pancreas

Generally, the diagnosis of ADP depends on combination of imaging examinations, including CT and ERCP or magnetic resonance cholangiopancreatography by demonstrating the absence of the pancreatic body, the tail and the missing duct of Santorini as well.^[32]^ ERCP is an invasive procedure and operator-dependent for successful identification of opacity of the main and accessory pancreatic duct. By contrast, magnetic resonance cholangiopancreatography has its advantage by clearly presenting the pancreatic duct morphology noninvasively. Ultrasound has limitations because of nonvisualization of the body and tail of the pancreas due to the interference from bowel gas or technical failure.^[33,34]^ Although the application of endoscopic ultrasound in the diagnosis of ADP has been proved to be effective and reliable.^[19]^ In the present case, CT scanning and ERCP was firstly performed preoperatively, and surgery finally confirmed the diagnosis of ADP by revealing the absence of the body and tail of the pancreas.

The differential diagnosis of ADP includes pancreatic fat replacement and distal pancreatectomy. The former occurs because of the atrophy of the distal pancreatic parenchyma, and MR is helpful to make the differential diagnosis by demonstrating the different fat signal of the head of the pancreas.^[18]^ In the cases associated with the absence of splenic vein and relevant operation history, distal pancreatectomy should be considered.^[35]^ Treatment is not necessary for asymptomatic ADP patients. The main goal of the therapy for ADP is to relieve the associated symptoms. A low-fat diet and diabetes control are recommended for the patients of ADP with DM.^[36]^ If the ADP patients have pancreatitis, pancreatic enzymes could be administrated to reduce the pancreatic secretion and promote pain relief.^[37]^ When pancreatic tumors or other malignant medical problems are suspected in ADP patients, surgical therapy like pancreaticoduodenectomy, even total pancreatectomy should be taken into consideration. Moreover, pancreatin supplement could be useful in ADP patients complicated with the symptoms of exocrine pancreatic insufficiency.^[18]^

The present ADP case presented with chronic pancreatitis, with moderate hyperplasia in focal ductal epithelium of the pancreas, which was not reported before. The treatment in this patient was equivalent to total pancreatectomy.

To summarize, ADP is a quite rare congenital malformation of the pancreas associated with abdominal pain, DM, or other diseases. Reported cases of ADP are rare. Therefore, in-depth investigations should be performed, for appropriate management.

## Acknowledgments

The authors thank the studies which were included in this literature review.

## Author contributions

LX and XB designed the research, summarized the present case and wrote the manuscript and contributed equally to this work; NM, PJ, FY and YZ reviewed the literatures and conducted the data search; NM conducted the follow up of the present case and assisted in editing the manuscript and the figure. All authors read and approved the manuscript.

**Data curation:** Leizhou Xia, Xuefeng Bu, Pengcheng Jiang, Feng Yu, Nana Meng.

**Funding acquisition:** Leizhou Xia.

**Investigation:** Leizhou Xia, Xuefeng Bu, Pengcheng Jiang, FengYu, Yongjun Zhang.

**Writing – original draft:** Leizhou Xia.

**Writing – review & editing:** Nana Meng.
